# Supplementary material for: Semantic embeddings reveal and address taxonomic incommensurability in psychological measurement
Source: Nat Hum Behav. 2025 Mar 11;9(5):944–54. doi: 10.1038/s41562-024-02089-y (PMC12106064; doi:10.1038/s41562-024-02089-y)
Supplement: Supplementary file 1 — Text and Supplementary Figs. 1–10. [file 41562_2024_2089_MOESM1_ESM.pdf]

nature human behaviour

Supplementary information https://doi.org/10.1038/s41562-024-02089-y

Semantic embeddings reveal and address taxonomic incommensurability in psychological measurement

In the format provided by the authors and unedited

Supplementary Materials for
Using embeddings to automate jingle–jangle detection and tackle taxonomic incommensurability

Dirk U. Wulff[1,2] and Rui Mata[1]
[1]Max Planck Institute for Human Development
[2]University of Basel

S1: Fine-tuning MPNet with personality data

Our approach is inspired by the work of Hommel and Arslan (2024), which has shown that a fine-tuned model can produce better results than off-the-shelf pre-trained models for the prediction of item similarity. Using personality data, we fine-tuned MPNet (Song et al., 2020), a lightweight transformer model based on the BERT architecture. MPNet belongs to the class of sentence-embedding models that can be used to generate numerical representations for whole sentences. Fine-tuning the MPNet model means providing new examples to adapt the embeddings it produces to the specific domain. Our model differs from that introduced by Hommel and Arslan (2024) in a number of respects, most prominently by aiming to produce a model to predict item relatedness irrespective of its direction (i.e., positive vs negative correlation between items).

We used data from three sources to perform model fine-tuning. First, we used data from the Open-Source Psychometrics Project, a citizen-science project gathering personality ratings via openpsychometrics.org. We used the data from four inventories: The 16PF inventory, consisting of 162 items and 16 scales; the BIG5 inventory, consisting of 50 items and 5 scales; the FFM inventory, consisting of 50 items and 5 scales; and the HEXACO inventory, consisting of 240 items and 24 scales. We restricted the data to US respondents only, resulting in 23,988 (16PF), 19,719 (BIG5), 546,403 (FFM), and 15,017 (HEXACO) respondents for the four inventories. Second, we included data from Kajonius and Johnson (2019, www.psycharchives.org/en/item/e42a4531-1daa-4f3d-aef4-58f085c77cd8), who conducted a large-scale citizen-science assessment of the NEO inventory consisting of 300 items and 30 scales. We used the data from 212,625 US respondents. Third, we included data from a subset of the data of the Eugene-Springfield Community Sample (L. Goldberg & Saucier, 2008, dataverse.harvard.edu), including items from the NEO and BIG5, as well as from 11 other personality inventories, such as the AB5C or JPI. The data of this subset consisted of 966 items and 1,142 respondents. In contrast to other sources, the data of the Springfield Community study has the advantage of providing information about cross-inventory relationships between items.

We aimed for a training set size of 200,000 examples, which we deemed sufficient to achieve successful domain adaption while keeping the computational load within feasible limits. The examples consisted of pairs of items and their unsigned (absolute) correlation as a measure of item relatedness. Together, the data from the Open-Source Psychometrics Project and Kajonius and Johnson (2019) permitted the creation of a total of 89,021 examples. The remaining 110,979 examples were sampled from the Springfield Community data proportionally to the unsigned correlations to slightly increase high correlation examples, which are rare, especially in the Springfield Community data. We processed the texts in the examples slightly to minimize systematic differences between data sources. First, consisting of IPIP wordings, we removed the "I" in front of many items. Thus, an item would read, for instance, "Have excellent ideas." instead of "I have excellent ideas." which was the most common format used in other data sources. Second, we ensured all item texts ended with a period. These changes helped ensure that no superficial aspects of the item texts influenced model predictions.

We fine-tuned MPNet using the sentence transformers library (Reimers & Gurevych, 2019). We adopted AdamW (Loshchilov & Hutter, 2017) as the optimizer and a linear warm-up for the first fifth of all batches of 64 examples. As the loss function, we used the `CosineSimilarityLoss` function in the `sentence_transformers` library. This function computes the Euclidean distance between the cosines predicted by the model and the criterion, in this case, the empirical absolute Pearson correlations between personality items. We fine-tuned the entire model.

We evaluated the model's out-of-sample performance for the five individual inventories to demonstrate successful domain adaption. It is common that inventories share a subset of items, implying a significant risk of data slippage, so we address this issue by removing those items from the training data. In each case, we trained the model on all examples that contained no items of the target inventory. For instance, to evaluate the performance in predicting the 1,225 examples of the FFM, we trained only on 168,265 examples, which is less than the remaining examples because 30,510 exam-

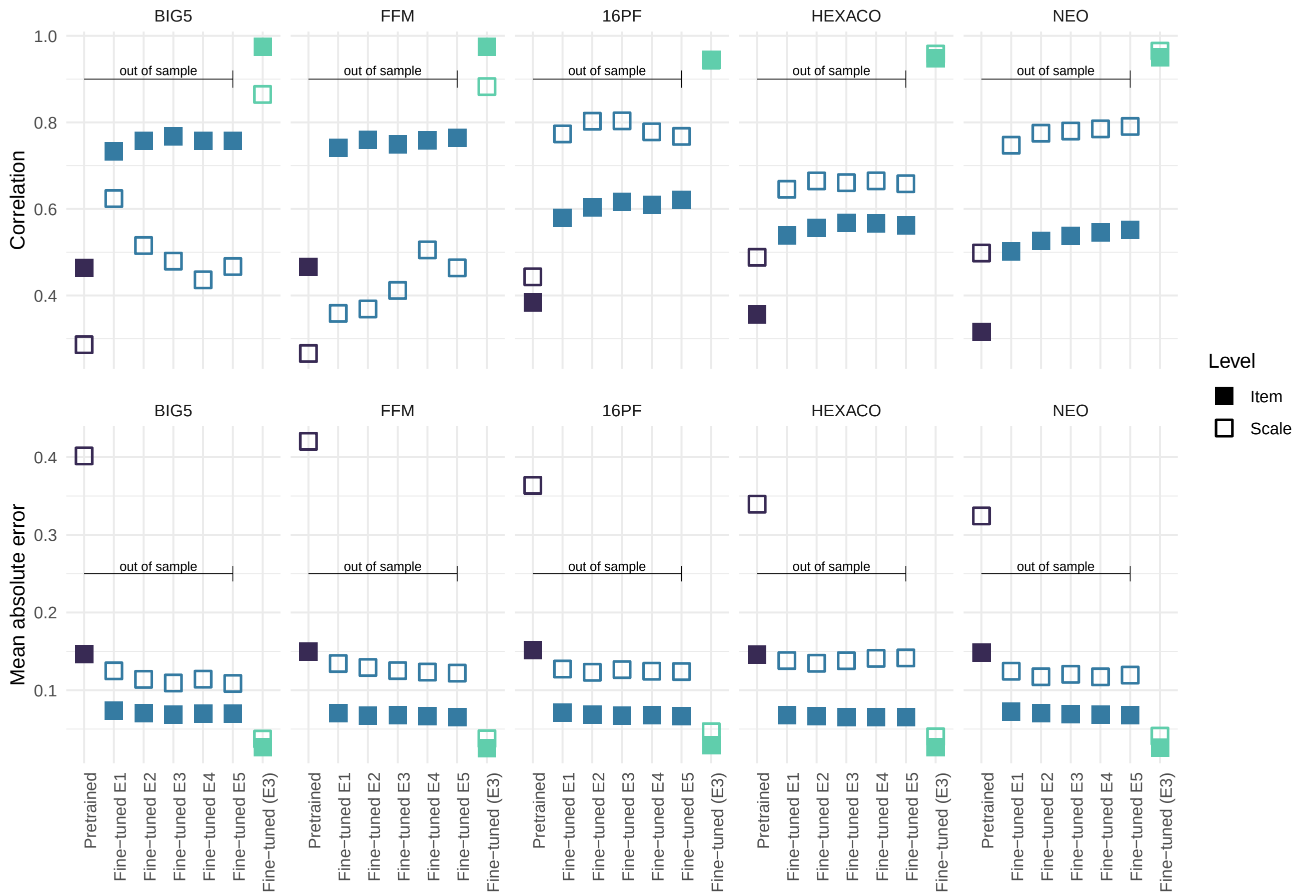

Supplementary Figure 1

Fine-tuning performance. The figure shows the performance of the pre-trained and fine-tuned MPNet in terms of the correlation and mean absolute error between predicted and observed item (full squares) and scale (open squares) correlations. The out-of-sample results (blue) are displayed by training epochs reflecting different stages of training. The in-sample performance (green) is shown for Epoch 3.

ples from other inventories included items from the FFM. We evaluated performances both at the item and scale level. Scale embedding being generated according to

$$e_j^{scale} = \frac{1}{n}\sum_j e_{ij}^{item}, \tag{1}$$

with e_j^{scale} being the embedding of the j-th scale and e_{ij}^{item} being the embedding of the i-th item in scale j.

The results displayed in Figure 1 show the out-of-sample performance after different amounts of training (Epochs) at the item and scale level, compared to the pre-trained model and a model trained on all examples (Full model). The figure shows that training substantially improves performance. At the level of items, performance rises from, on average, $r = 0.40$ to $r = 0.65$, with most of the performance increase being realized by Epoch 3 ($r = 0.65$). At the scale level, performance was similarly boosted from $r = 0.40$ to $r = .63$. Scale-level out-of-sample performance was lower than item-level performance for two inventories (BIG5 and FFM) and higher for the other three (16PF, HEXACO, NEO). These lower results for the BIG5 and FFM can be explained by the small number and character of the scales, which in these cases are factors that were trained to be mostly orthogonal, implying a significant reduction in variance. The higher performance for the 16PF, HEXACO, and NEO can be explained by a less extreme skewness of the scales than for items due to their much larger numbers. Note that, for any inventory, the number of conceptually unrelated items grows quadratically, whereas the number of conceptually related items grows only linearly. Thus, the distribution of 30 subscales in the case of, for instance, the NEO, is much less skewed than that of its 300 items. Overall, analyzing the results using mean absolute error reveals that the performance on items, on average $MAE = 0.068$, is systematically (for all

inventories) better than the performance on scales, on average $MAE = 0.12$.

Finally, the full model shows extremely high performances at the item ($r = 0.96$, $MAE = 0.027$) and scale ($r = 0.93$, $MAE = 0.040$) levels. This level of performance is not surprising given this has been given access to all correlations during training, but it is nevertheless important. It demonstrates that MPNet can discriminate between the meanings of items and memorize a large of empirical correlations. As a result, this model represents an accurate substitute for empirical correlations. It will perform extremely well on known examples and generalize roughly at the same level as the out-of-sample performance shown in 1.

S2: An overview of language model competitors

We consider several competitors to our fine-tuned MPNet language model. Table 1 provides an overview. We selected these models because they cover a wide range of approaches that can be used to generate embeddings. In addition to the base and fine-tuned MPNet, we consider additional transformer models: the Instructor (XL), a 1.5 billion parameter powerful open-source model, and an embedding model from OpenAI. Being built on the transformer architecture, these models produce embeddings for entire texts and are sensitive to information concerning word order, semantic context, and other syntactic information. Furthermore, we considered two word-based models: fastText, a popular word embedding model (Mikolov et al., 2017), and LSA, a model previously proposed to evaluate the relatedness of personality scales (Rosenbusch et al., 2020).

The models differ in how they can be used to generate embeddings. For all of the sentence-embedding models, we directly retrieved embeddings from publicly available application programming interfaces (API) or repositories (see final column in Table 1). For the fastText word-based embeddings, we generated embeddings according to

$$e_j^{item} = \frac{\sum_i n_i \cdot e_{ij}^{word}}{||\mathbf{n}||_2} \tag{2}$$

where e_{ij}^{word} is the embedding of the i-th distinct word and n_i the frequency of word i in the text of an item j. Finally, we obtained embeddings from the latent semantic analysis approach following Rosenbusch et al. (2020) by first stemming the tokens in the text and then employing singular value decomposition to obtain the 300 components that best account for the distribution of tokens across items or scales.

We implemented two ways of obtaining embeddings at the level of personality scales from each model. The first way was to average the embeddings of all items that compose a scale according to the formula above. The second way (*Scale (concat)*), inspired by (Rosenbusch et al., 2020), was to concatenate the items belonging to a scale into a single unit text and then to retrieve embeddings for the scale-level texts directly.

Finally, we also consider the Construct Identity Detector (CID) model developed by Larsen and Bong (2016). However, as the CID model is not a traditional embedding model, we evaluate it separately and present the results in section S6.

S3: Evaluating the suitability of embeddings to recover psychometric characteristics of psychological measures

Our goal is to explore how different embedding models allow us to understand the relation between personality measures, particularly the extent to which embeddings match past insights regarding the intercorrelations between items or scales. Please note that our model comparisons focus on two units of analysis: items and scales. One reason for considering both items and scales is that different applications may profit from relying on these different units. For example, if one is interested in identifying new items for a novel measure, it may be helpful to have a tool that provides item similarity of the new item to extant ones in the literature. In turn, if one is interested in estimating the empirical link between constructs, focusing on scales may be more adequate because the latter are typically used to measure constructs. The issue of unit is also relevant to the model selection issue because model evaluation can differ depending on the input used and the associated choices in preprocessing these inputs (item vs. scale). Consequently, we consider both items and scales and different preprocessing options in our validations. In what follows, we specifically evaluate the ability of item and scale embeddings to predict (a) internal consistency, (b) factor structure, (c) convergent validity, and (d) divergent validity of personality measures. These results can be seen as different forms of validation and, as a whole, provide an overview of the current power of different language models to reveal the structure of human personality.

Internal Consistency

Internal consistency refers to the extent to which all items of a measure capture the same construct and is usually assessed as the interrelatedness of the items of a given measure; in psychology, Cronbach's α is a common way to assess the inter-item reliability, or internal consistency, of a set of items.

We were interested in determining to what extent similarity from word and sentence embeddings can be used to identify the internal consistency of personality measures. For this purpose, we used the average cosine similarity obtained from embeddings for items of a given scale to predict the scale's internal consistency as estimated from previous self-reports of personality (i.e., Cronbach's α). We used the Spearman–Brown prediction formula that calculates the expected internal consistency of a scale based on a scale's average item correlation and the number of items. Empirical values of Cronbach's α were taken from IPIP (http://ipip.ori.org;

Table 1
Models

Label	Description	Reference	Source
MPNet	Open-source encoder transformer model (all-mpnet-base-v2) based on BERT producing text embeddings	Song et al. (2020)	Huggingface
MPNet-ft-is	Open-source encoder transformer model based on BERT producing text embeddings	see previous section	Huggingface
MPNet-ft-oos	Open-source encoder transformer model based on BERT producing text embeddings	see previous section	Huggingface
mixedbread	Open-source encoder transformer model (mxbai-embed-large-v1) producing text embeddings	Lee et al. (2024)	Huggingface
OpenAI	Latest proprietary model (text-embedding-3-large) from OpenAI producing text embeddings	Zhuang et al. (2024)	Open AI API
fastText	Open-source bag-of-words neural network producing word embeddings	Mikolov et al. (2017)	fastText
LSA	Natural language processing approach using singular value decomposition applied to a matrix tabulating the co-occurrences of documents (items or scales) and tokens (stemmed words); produces word and text embeddings	Rosenbusch et al. (2020)	Own computations

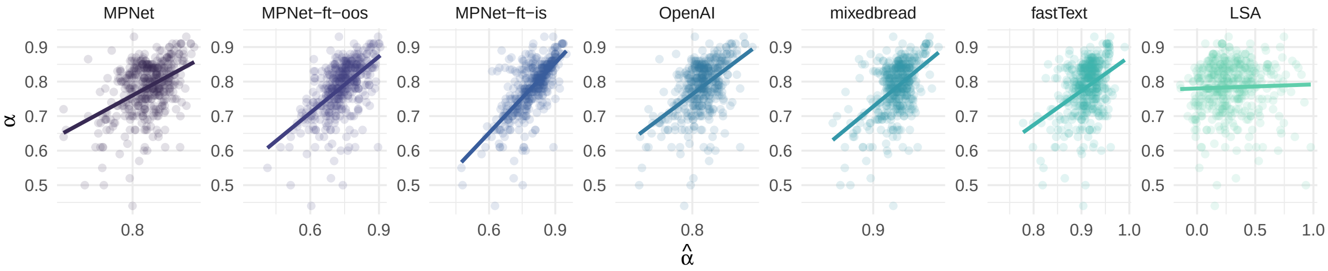

Supplementary Figure 2
Internal consistency. The figure shows correlations between the predicted internal consistency obtained from eight models and the observed internal consistency, Cronbach's α, for 459 scales in IPIP. The predictions were based on either the original item text (top row) or the preprocessed item text with no stop-words (bottom row).

L. R. Goldberg et al., 2006). Overall, values were available for 448 of the 459 scales considered. Where multiple values were available, we took the average.

Figure 2 shows the correlation for the different models. It can be seen that all models except LSA could recover scale internal consistencies reasonably, with performance for these models ranging between r = .70 and r = .40. The best three performing models were MPNet-ft-is (r = .70), MPNet-ft-oos (.57), and OpenAI (r = .51). LSA's performance was considerably lower with r = .03.

Factor Structure

Personality inventories are typically designed to measure several constructs and will contain several scales, each composed of multiple items corresponding to the same construct or factor. As a second form of validation, we evaluated whether the item similarities implied by embeddings tend to reflect the factor structure implied in the respective inventories. Specifically, we evaluated whether the average similarity between items of the same scale is higher than between items of different scales within a given inventory. To the extent that items are assigned to factors based on their similarity as determined by word or sentence embeddings, we can think of this assignment as the ability of embeddings to capture the inventory's factor structure.

Figure 3 shows the average cosine similarity within a given scale z-standardized relative to the similarity between items of that scale and other scales. Values above 2 indicate that the within-scale similarity is two standard deviations higher than between-scale similarities, implying a good recovery of factor structure for that scale. The analysis reveals that most models well-recovered the factor structure implied for a large fraction of scales, with rates ranging between 13.5% and 100%. The top three models were MPNet-ft-oos (100%), MPNet-ft-is (97%), and OpenAI (85%).

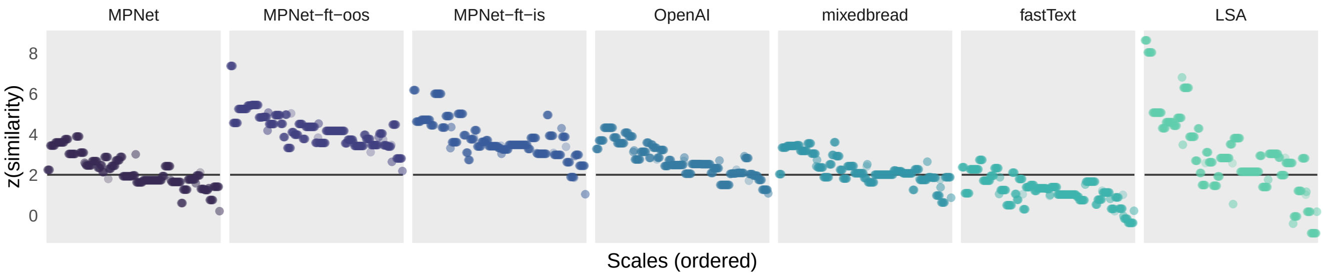

Supplementary Figure 3

Factor structure. The figure shows the standardized similarity between items belonging to a given scale relative to the similarity between items of that scale and those of other scales. Scales are ordered by the median relative similarity across models.

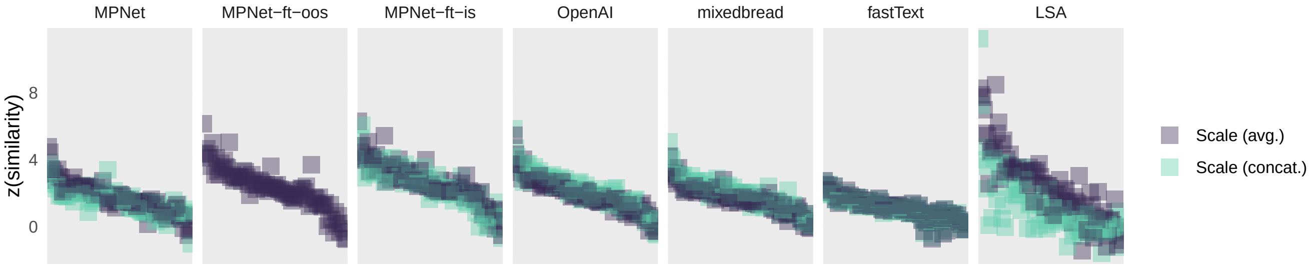

Supplementary Figure 4

Convergent validity. The figure shows the standardized similarity between scales belonging to the same label relative to the similarity between scales of different labels. Constructs are ordered by the median relative similarity across models. Panel A shows the results for item-base scale models, and panel B the results of scale-based models.

Convergent validity

Convergent validity refers to how closely a scale is related to other measures of the same construct. To evaluate convergent validity, we analyzed the similarity of scales that represent the same construct as reflected in the scale's name in the IPIP database. We focused on 49 construct labels present at least three times in IPIP (e.g., extraversion, trust) and analyzed whether the similarity between scales with the same labels exceeds the similarity of scales with different labels. For this analysis, and as described in the methods section above, we constructed scale-based embeddings by (a) averaging item-based embeddings according to equation 1 or (b) by deriving embeddings directly for the scale's full text consisting of the concatenated texts of individual items.

Figure 4 shows the relative similarity between scales of the same given label versus the similarity between scales of the label and other labels, such that values above 2 indicate good recovery of convergent validity. We observed that the models varied in how well they recovered the convergent validity between scales of the same label, with results ranging from 6.1% to 91.8%. The top three models were MPNet-ft-is (avg.: 91.8%; concat.: 77.6%), MPNet-ft-oos (avg.: 69.4%), and OpenAI (avg.: 51%; concat.: 63.3%). There were no systematic differences between averaged and concatenated scale embeddings.

Divergent validity

Divergent validity refers to the idea that a measure should not correlate with other measures that are dissimilar or unrelated to the measure of interest. We evaluated divergent validity using five data sets used above to fine-tune MPNet. We used Pearson correlation to compare the empirical correlations against the predicted correlations based on item and scale similarities.

Figure 5 shows the correlations between observed and predicted correlations for item and scale-based models. The models' average ability to capture the empirical scale correlations varied from r = .0.07 and r = .86. The best three models were MPNet-ft-is (item: r = .96, scale avg.: r = .92, scale concat.: r = .71), MPNet-ft-oos (item: r = .65, scale avg.: r = .63), and OpenAI (item: r = .50, scale avg.: r = .53, scale concat.: r = .32).

Overall assessment

Our evaluation has shown that embedding models can accurately capture key patterns of empirical personality data. The best model overall was the MPNet model fine-tuned on all data (MPNet-ft-is). This result is unsurprising, given the overlap between the training and evaluation data. Nevertheless, it showcases the ability of MPNet to accurately fit the

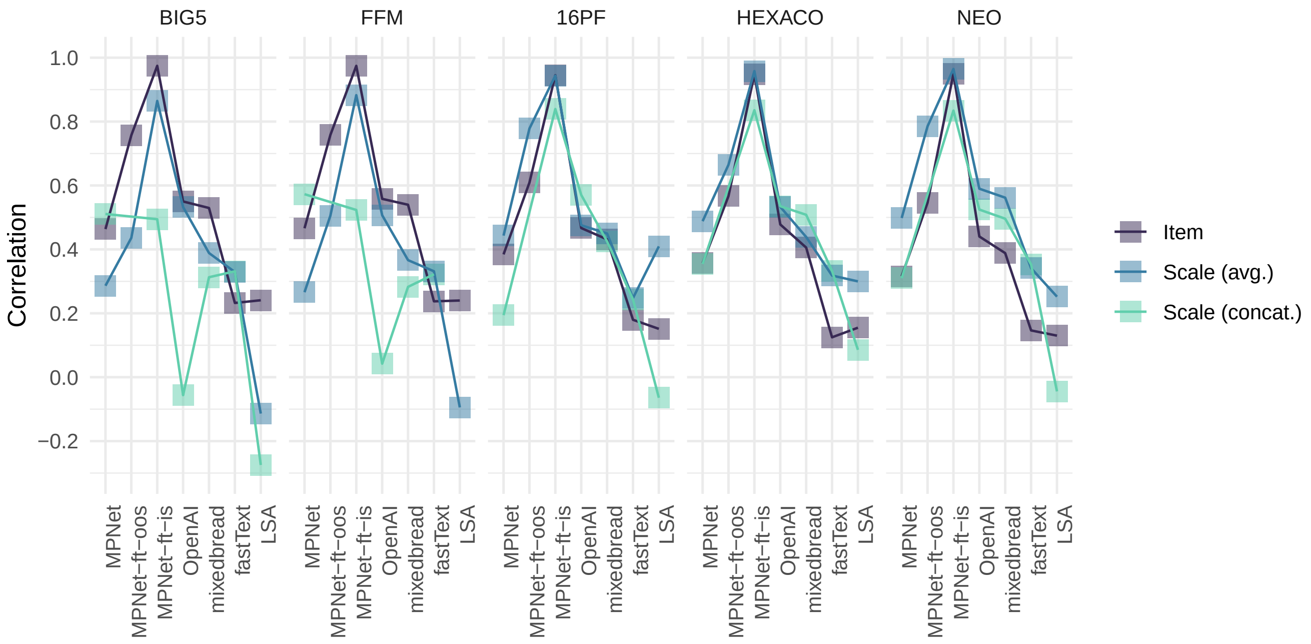

Supplementary Figure 5

Divergent validity. The figure shows the Pearson correlations between the predicted and observed correlations for the items and scales of five personality inventories. See section Fine-tuning MPNet with personality data.

personality data. Crucially, the next best model was the fine-tuned MPNet model evaluated out-of-sample, outperforming the third-best model from OpenAI in all evaluation steps. This demonstrates that the fine-tuned MPNet model not only fits personality nearly perfectly but also generalizes better than any competitor model. Consequently, the fine-tuned MPNet model is the best replacement for empirical empirical data.

S3: Evaluating construct label definitions

To be able to identify jingle–jangle fallacies and produce new mappings of psychological constructs and their measures, it is necessary to evaluate the embeddings not only of personality items and scales but also those of the respective construct labels. First, one simple way to do this is to embed the construct labels (e.g., "Extraversion" or "Sociability") to represent the construct. However, because many construct labels can be used in everyday language without explicit reference to the personality construct (e.g., "Intellect" or "Warmth"), additional context could be helpful to capture the meaning of the construct. Therefore, in what follows, we introduced additional ways to provide context. Second, we produced a contextualized version of the construct labels by placing the construct label in the sentence "The personality construct [LABEL]." in place of [LABEL]. Third, we scraped definitions of the available personality constructs from the APA dictionary (https://dictionary.apa.org/). Construct definitions were available for 135 of a total of 277 distinct constructs. Fourth, we manually curated the APA definitions by removing parts of the definitions that refer to meanings other than the personality construct (e.g., reference to special meanings of the construct "Competence" in linguistics and law) and filling in text for constructs that are defined exclusively by providing reference to other constructs using the definitions of the other constructs (e.g., replacing "See aggression" with the definition of "Aggression" for the construct "Hostile-Aggression"). Fifth, we generated definitions from GPT-4 via the API using a variety of different prompt structures varying the length (30, 50, or 100 words), assistant instruction (e.g., starting or not starting the prompt with "You are an expert in psychology and will be asked to produce a definition for a construct that other experts in the field will recognize as accurate and representative."), and core prompt ("Write a [LENGTH]-word expert definition of the personality construct [LABEL]." or "Write a [LENGTH]-word expert definition of the personality construct [LABEL].").

We evaluated embeddings of the labels, the contextual labels, APA definitions, and GPT-4 definitions by computing the similarity of construct and scale embeddings. Specifically, we computed an z-score for each scale i and construct embedding model k as $z_i k = \frac{cos_{ijk} - c\bar{o}s_i k}{sd_{cos_{ik}}}$, where cos_{ijk} is the cosine similarity between scale i and its corresponding construct j given embedding model k and $c\bar{o}s_i k$ and $sd_{cos_{ik}}$

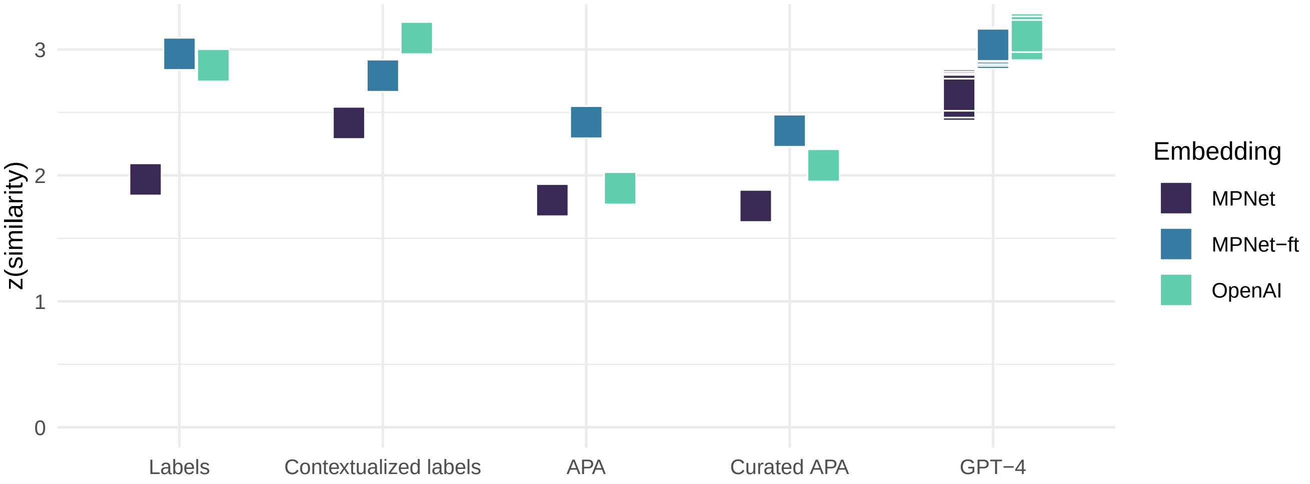

Supplementary Figure 6

Construct label evaluation. The points show the average alignment score as defined in the text for embeddings of the constructs implied by IPIP labels, contextualized labels, APA definitions, curated APA definitions, and definitions generated by GPT-4 for two embeddings (ADA, Instructor).

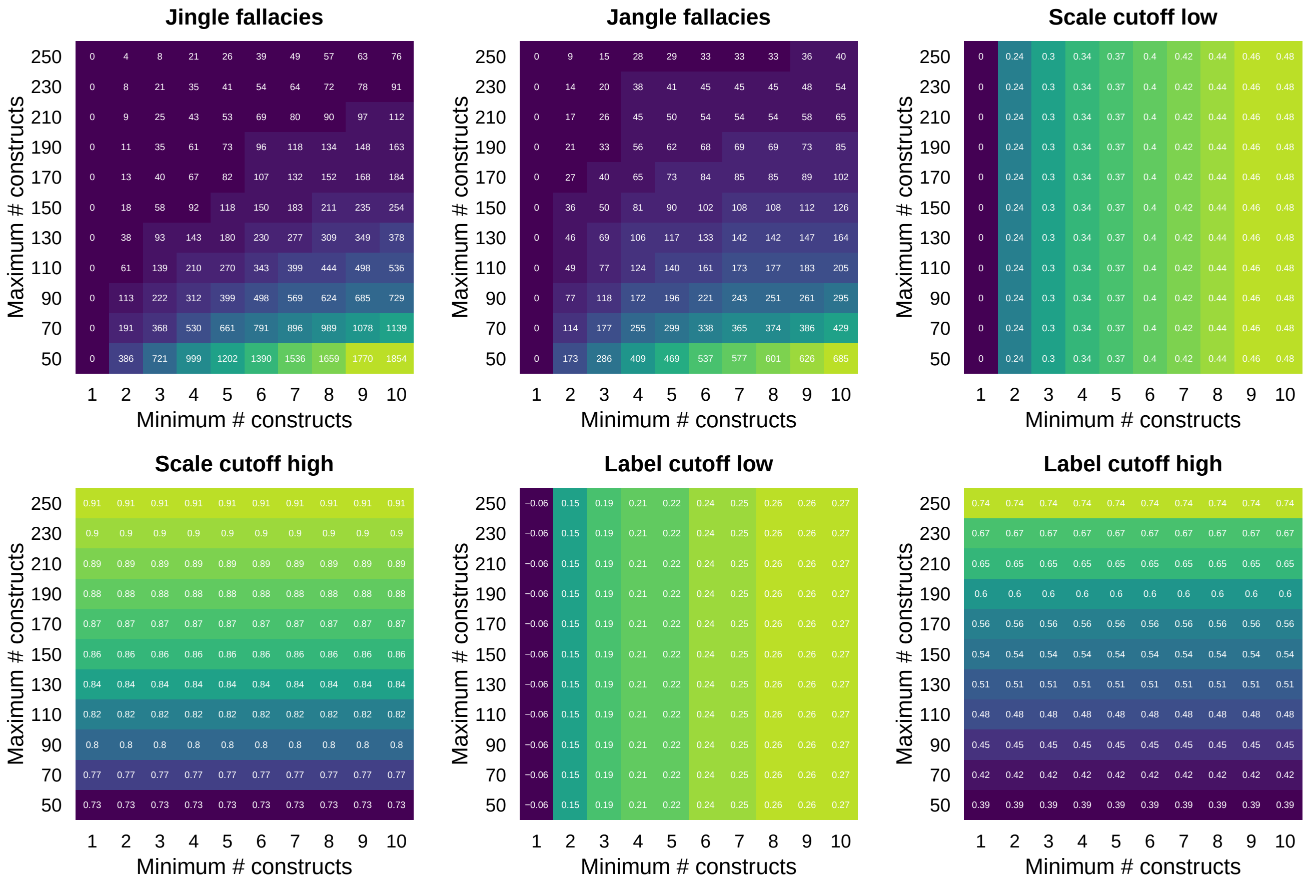

Supplementary Figure 7

Jingle–jangle thresholds. The first two panels show the number of jingle and jangle fallacies implied by different similarity cutoffs expressed as the assumed minimum and maximum number of constructs. The following four panels show the actual cosine similarity thresholds to assert that two scales or labels are distinct (low threshold) or the same (high threshold).

the corresponding means and standard deviations across all constructs. We then averaged the scores across scales to obtain an overall estimate of how well a given instantiation of labels is aligned with the corresponding scales. We computed the average z-score for a subset of embedding models, which emerged as best to capture empirical relationships between items and scales. Figure 6 shows the results for the labels and additional contenders. The original labels showed high scores of 1.97 (MPNet), 2.96 (MPNet-ft-is), and 2.87 (OpenAI), implying that, on average, the similarities between scale and matching construct embeddings were two to three standard deviations higher than those between scale and non-matching construct embeddings. This alignment was improved slightly for some models by using contextualized labels (MPNet: 2.42; MPNet-ft-is: 2.79; OpenAI: 3.09) and the GPT-4 definitions (MPNet: 2.64; MPNet-ft-is: 3.01; OpenAI: 3.09), who both showed about equally good alignment. By contrast, the original APA definitions (MPNet: 1.80; MPNet-ft-is: 2.42; OpenAI: 1.90) and the curated APA definitions (MPNet: 1.76; MPNet-ft-is: 2.35; OpenAI: 2.08) showed significantly lower scores. Some of the score reduction for the APA definitions can be explained by the fact that only a portion of scales and constructs can be considered due to missing definitions. However, even when constrained to the available data, the labels and contextualized labels showed substantially higher scores.

The alignment scores suggest that constructs can be captured well using the construct and that there is little to gain by contextualizing the constructs or using AI-generated definitions. Moreover, although the OpenAI model showed overall the highest scores, the fine-tuned MPTNet model was a very close second and outperformed the OpenAI for the labels. Consequently, we decided to rely on the labels and the fine-tuned MPTNet for our jingle–jangle and relabeling analyses.

S4: Evaluating the robustness of jingle–jangle analyses

Jingle-jangle criteria

Identifying jingle and jangle fallacies strongly hinges on the thresholds used to assert that two scales or labels are distinct or identical. In our main analysis, we have chosen a threshold based on principled reasoning concerning the minimum and maximum number of constructs needed to capture the items in IPIP. Here, we analyze the consequences of using other thresholds. As can be seen in Figure 7, the number of jingle and jangle fallacies vary dramatically as a function of thresholds, as one would expect. For lenient thresholds (i.e., higher minimum number of constructs and lower maximum number of constructs), the number of jingle and jangle fallacies are both high, whereas, for conservative thresholds, both are low. This illustrates that there is no correct number or true number of fallacies and that numbers depend on how lenient or strict the thresholds are for identifying scales or constructs as distinct or identical. Nevertheless, across most threshold values, jingle fallacies appear to be more frequent than jangle fallacies. This suggests that the assignment of scales to constructs is a bigger problem than the labels given to these construct groups.

Jingle-jangle minimization

We evaluated a number of different clustering algorithms using our fine-tuned model to obtain mappings between scales and labels that minimize jingle–jangle fallacies. Specifically, we considered *hierarchical clustering* with *complete*, *ward.D*, and *single* linkage, as well as *Kmeans* and *Mclust* (Gaussian mixture) clustering. Figure 8 shows the trade-off between the number of jingle and jangle fallacies for 1 to 250 different clusters. It can be seen that the choice of clustering algorithm has a considerable effect on the number of clusters needed to minimize jingle and/or jangle fallacies. This effect is due to the different optimization criteria employed by the algorithms. For instance, hierarchical clustering with complete linkage seeks to minimize the maximum distance between objects within clusters, whereas hierarchical clustering with single linkage seeks to maximize the minimum distance between clusters. This directly influences the number of jingle and jangle fallacies they commit, with complete linkage primarily minimizing jingle fallacies and single linkage primarily minimizing jangle fallacies. The other algorithms generally seek to maximize between or within-cluster variance and fall, for this reason, between the other two algorithms. Despite these differences, all clustering algorithms except hierarchical clustering with single linkage produce solutions that use fewer clusters than implied by IPIP while still reducing the number of fallacies.

Overall, these results underscore the power of clustering personality measures using different high-performing large language models to minimize one or both types of fallacy.

S5: Extending jingle-jangle detection and minimization to data from Rosenbusch et al. (2020)

One question concerning our analysis of jingle-jangle fallacies in the IPIP database is which patterns generalize to other data sets of psychological measures and which might be unique to IPIP. To assess this, we generalized our approach to a large database gathered by Rosenbusch et al. (2020), which the first author kindly provided to us. The database consists of 38,298 items, 2699 scales, and 2470 construct labels. To prepare this data for analysis, we homogenized the item and label texts by removing superfluous punctuation, numbering, and blank spaces. We then embedded the item and label text using the fine-tuned MPNet model and determined fallacy thresholds using the same quantile-based approach as applied to IPIP. Figure 9 shows the number of jingle and jangle fallacies as a function of different thresholds. It can be seen that, as with IPIP, the thresholds

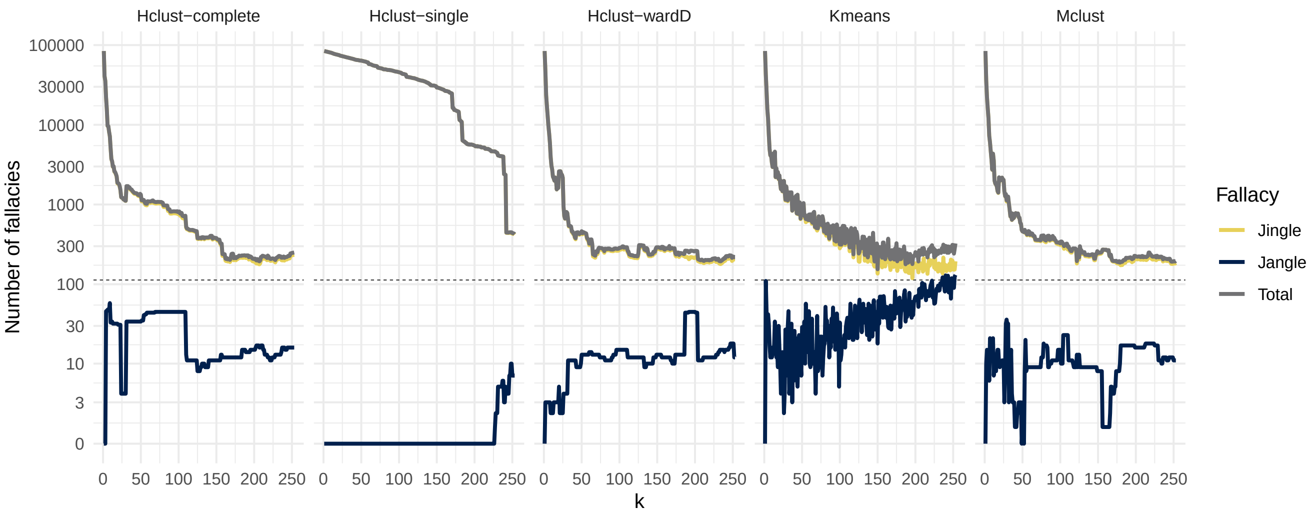

Supplementary Figure 8

The jingle–jangle trade-off for different clustering approaches. The panels show the individual number and sum (gray line) of potential jingle (yellow line) and jangle (blue line) fallacies committed by different clusterings, varying the number of clusters (x-axis), the embedding (rows), and clustering algorithms (columns).

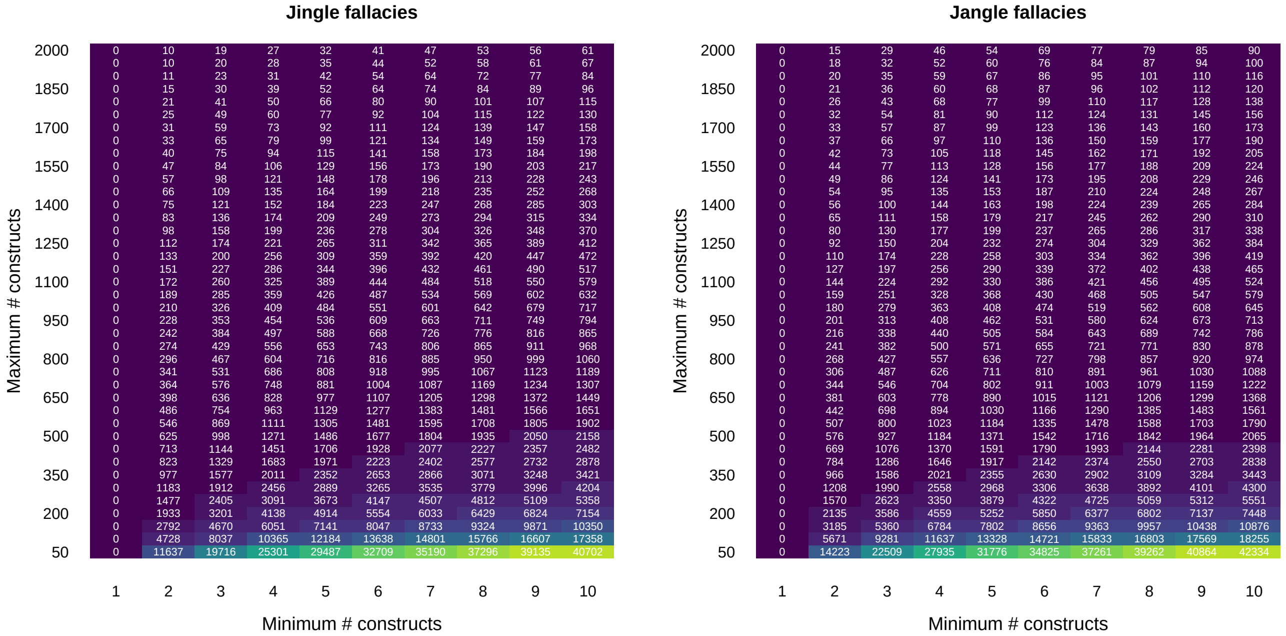

Supplementary Figure 9

Jingle–jangle thresholds for the Rosenbusch et al. (2020) data. The panels show the number of jingle and jangle fallacies implied by different similarity cutoffs expressed as the assumed minimum and maximum number of constructs.

strongly influence the number of fallacies, with more lenient thresholds leading to more fallacies and vice versa.

Using the same threshold values of 5 and 100 as used for IPIP, we observed 12,184 jingle and 13,328 jangle fallacies, where using a maximum number of constructs (900) reflecting the same as 100 does for IPIP, we observed 588 jingle and 505 jangle fallacies. These results again highlight the important influence of thresholds. Overall, we observed more jingle fallacies than jangle fallacies for 59% of threshold levels, matching the relative frequencies of jingle and jangle found for IPIP. Furthermore, the total number of fallacies relative to the total number of scales was roughly comparable between

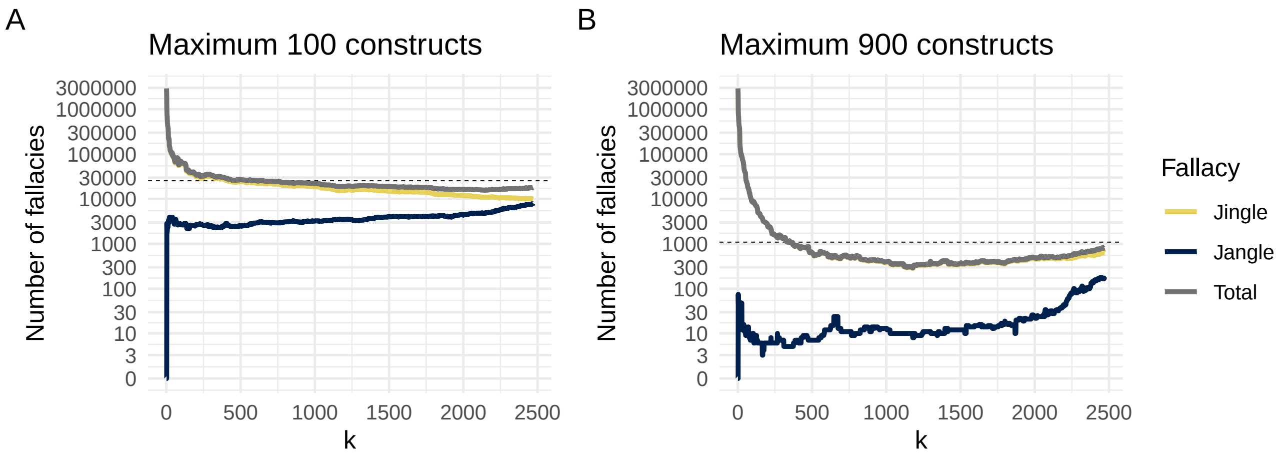

Supplementary Figure 10

The jingle–jangle trade-off for Rosenbusch et al. (2020). The plot shows the individual number and sum (gray line) of potential jingle (yellow line) and jangle (blue line) fallacies committed by hierarchical clustering with Ward linkage, varying the number of clusters. Panels A and B show the results for thresholds assuming a maximum number of constructs of 100 and 900, respectively.

the Rosenbusch et al. data (.7%) and IPIP (.5%) when using the same 5/100 thresholds.

Figure 10 shows the results of our relabeling method, using hierarchical clustering with *wardD* linkage, for different numbers of clusters. Analog to the results for IPIP, jingle fallacies decline for increasing numbers of clusters while jangle fallacies increase, replicating the jingle-jangle tradeoff observed for IPIP. Furthermore, the relabeling method produced parsimonious solutions with as few as 607 clusters that committed fewer fallacies than the 2470 clusters implied by the original mapping of scales and labels. Using the stricter criterion of 900 constructs, as few as 337 clusters were sufficient to produce fewer fallacies than the original mapping. Optimal solutions committed 15,712 fallacies (38% reduction; 100 construct threshold) and 292 (73% reduction; 900 construct threshold) with 2,140 and 1,178 clusters, respectively.

Despite the similarities in results, it is important to point out that there are significant differences between the IPIP and the Rosenbusch et al. (2020) data. First, the level of curation of item and label texts is higher in IPIP than in the Rosenbusch et al. data. Second, the data from Rosenbusch et al. (2020) covers a much larger range of constructs beyond the core personality ones covered by IPIP, such as religious and political practices, eating behavior, or clinical diseases.

Two datasets containing the jingle-jangle classifications and the labels assigned during relabeling can be found in this repository: https://osf.io/nmv29/.

S6: Evaluating the Construct Identity Detector (CID) model

The Construct Identity Detector (CID) model proposed by Larsen and Bong (2016) implements a combination of natural language processing techniques, considering word vectors, word order, and word frequency, and has been found to outperform latent semantic analysis in predicting expert assessments of construct identity. CID is not a traditional embedding model. Instead of mapping inputs into a common semantic space, CID constructs embeddings that are specific to each pair of inputs provided to the model.

We implemented the CID with two alterations, which we believe strengthened the model in comparison to the original implementation by Larsen and Bong (2016). First, instead of using LSA, we use the pretrained fastText word embedding model (https://fasttext.cc/docs/en/english-vectors.html) to determine word-word similarities. This model has been trained on hundreds of billions of tokens and has outperformed LSA in our analysis. Second, we use SUBTLEX-US (Brysbaert & New, 2009) instead of the Brown Corpus to determine word weights, because the former has been found to provide a better account of human evaluations.

We evaluate CID to other models concerning its ability to predict the correlation between items, which is the basis for being able to predict relationships between scales and between scales and labels and thus provides an important proxy for downstream evaluations. We found that CID predicts the absolute correlations between items well. Specifically, we found correlations ranging between r = 0.236 (NEO) and r = 0.350 (FFM) and between MAE = 0.139 (FFM) and

MAE = 0.158 (NEO). These values are higher than those observed for fastText alone or LSA, supporting the conclusion of Larsen and Bong that a combination of embeddings and other NLP approaches can be beneficial. However, these values are also substantially lower than those obtained for large language models. Our fine-tuned model achieved out-of-sample performance between r = 0.546 (NEO) and r = 0.758 (FFM) and between MAE = 0.065 (HEXACO) and MAE = 0.070 (BIG5), and the OpenAI large model, which was the best off-the-shelf model, achieved performances of r = 0.440 (NEO) and r = 0.558 (FFM) and between MAE = 0.118 (NEO) and MAE = 0.139 (FFM). The difference between the performance of CID and the large language models can be attributed to the crucial differences between traditional word embedding models and newer text embedding models. Newer text embedding models are based on the transformer architecture, which enables the embedding to interpret the meaning of words in the context of other words, leading to significantly improved evaluations of the similarity of texts.

References

Brysbaert, M., & New, B. (2009). Moving beyond Kučera and Francis: A critical evaluation of current word frequency norms and the introduction of a new and improved word frequency measure for American English. *Behavior Research Methods*, *41*(4), 977–990.

Goldberg, L. R., Johnson, J. A., Eber, H. W., Hogan, R., Ashton, M. C., Cloninger, C. R., & Gough, H. G. (2006). The international personality item pool and the future of public-domain personality measures. *Journal of Research in Personality*, *40*(1), 84–96. https://doi.org/10.1016/j.jrp.2005.08.007

Goldberg, L., & Saucier, G. (2008). The eugene-springfield community sample: Information available from the research participants. *Oregon Research Institute Technical Report*, *48*(1).

Hommel, B. E., & Arslan, R. C. (2024). Language models accurately infer correlations between psychological items and scales from text alone. https://doi.org/10.31234/osf.io/kjuce

Kajonius, P. J., & Johnson, J. A. (2019). Assessing the structure of the five factor model of personality (ipip-neo-120) in the public domain. *Europe's Journal of Psychology*, *15*(2), 260.

Larsen, K. R., & Bong, C. H. (2016). A tool for addressing construct identity in literature reviews and meta-analyses. *MIS Quarterly*, *40*(3), 529–551. https://doi.org/10.25300/MISQ/2016/40.3.01

Lee, S., Shakir, A., Koenig, D., & Lipp, J. (2024). Open source strikes bread - new fluffy embeddings model. https://www.mixedbread.ai/blog/mxbai-embed-large-v1

Loshchilov, I., & Hutter, F. (2017). Decoupled weight decay regularization. *arXiv preprint arXiv:1711.05101*.

Mikolov, T., Grave, E., Bojanowski, P., Puhrsch, C., & Joulin, A. (2017). Advances in pre-training distributed word representations. *arXiv preprint arXiv:1712.09405*.

Reimers, N., & Gurevych, I. (2019). Sentence-bert: Sentence embeddings using siamese bert-networks. *arXiv preprint arXiv:1908.10084*.

Rosenbusch, H., Wanders, F., & Pit, I. L. (2020). The Semantic Scale Network: An online tool to detect semantic overlap of psychological scales and prevent scale redundancies. *Psychological Methods*, *25*(3), 380–392.

Song, K., Tan, X., Qin, T., Lu, J., & Liu, T.-Y. (2020). Mpnet: Masked and permuted pre-training for language understanding. *Advances in Neural Information Processing Systems*, *33*, 16857–16867.

Zhuang et al. (2024). New embedding models and api updates.
