# Supplementary material for: Semantic embeddings reveal and address taxonomic incommensurability in psychological measurement
Source: Nat Hum Behav. 2025 Mar 11;9(5):944–54. doi: 10.1038/s41562-024-02089-y (PMC12106064; doi:10.1038/s41562-024-02089-y)
Supplement: Supplementary file 2 — Reporting Summary [file 41562_2024_2089_MOESM2_ESM.pdf]

nature portfolio

Corresponding author(s): Dirk Wulff

Last updated by author(s): 2024-

Reporting Summary

Nature Portfolio wishes to improve the reproducibility of the work that we publish. This form provides structure for consistency and transparency in reporting. For further information on Nature Portfolio policies, see our Editorial Policies and the Editorial Policy Checklist.

Statistics

For all statistical analyses, confirm that the following items are present in the figure legend, table legend, main text, or Methods section.

n/a	Confirmed	
☐	☒	The exact sample size (*n*) for each experimental group/condition, given as a discrete number and unit of measurement
☐	☒	A statement on whether measurements were taken from distinct samples or whether the same sample was measured repeatedly
☒	☐	The statistical test(s) used AND whether they are one- or two-sided<br>*Only common tests should be described solely by name; describe more complex techniques in the Methods section.*
☐	☒	A description of all covariates tested
☐	☒	A description of any assumptions or corrections, such as tests of normality and adjustment for multiple comparisons
☐	☒	A full description of the statistical parameters including central tendency (e.g. means) or other basic estimates (e.g. regression coefficient) AND variation (e.g. standard deviation) or associated estimates of uncertainty (e.g. confidence intervals)
☒	☐	For null hypothesis testing, the test statistic (e.g. *F*, *t*, *r*) with confidence intervals, effect sizes, degrees of freedom and *P* value noted<br>*Give P values as exact values whenever suitable.*
☒	☐	For Bayesian analysis, information on the choice of priors and Markov chain Monte Carlo settings
☐	☒	For hierarchical and complex designs, identification of the appropriate level for tests and full reporting of outcomes
☐	☒	Estimates of effect sizes (e.g. Cohen's *d*, Pearson's *r*), indicating how they were calculated

Our web collection on statistics for biologists contains articles on many of the points above.

Software and code

Policy information about availability of computer code

Data collection	No software was used for data collection.
Data analysis	The codes used to analyze the data are publicly available in an online repository (https://osf.io/nmv29/). Analyses were performed using the R programming language (version 4.3.1) and Python (version 3.9.16).

For manuscripts utilizing custom algorithms or software that are central to the research but not yet described in published literature, software must be made available to editors and reviewers. We strongly encourage code deposition in a community repository (e.g. GitHub). See the Nature Portfolio guidelines for submitting code & software for further information.

Data

Policy information about availability of data

All manuscripts must include a data availability statement. This statement should provide the following information, where applicable:

- Accession codes, unique identifiers, or web links for publicly available datasets
- A description of any restrictions on data availability
- For clinical datasets or third party data, please ensure that the statement adheres to our policy

Data used in our work (personality measures and data from IPIP and Rosenbusch et al., 2020) is made publicly available by the original data providers and the web links are directly provided in the manuscript. We make the curated data for IPIP labels and APA definitions available in our public repository (https://osf.io/nmv29/).

Research involving human participants, their data, or biological material

Policy information about studies with human participants or human data. See also policy information about sex, gender (identity/presentation), and sexual orientation and race, ethnicity and racism.

Reporting on sex and gender	We do not make use of sex or gender information in our analysis.
Reporting on race, ethnicity, or other socially relevant groupings	We did not include race or ethnicity in our analyses. However, in our validation of item and scale embeddings we use personality data for and English-speaking US-based group because of our focus on English-based embeddings of personality items and construct definitions.
Population characteristics	We use personality data from a US sample and several citizen-science data sets from the Open Psychometrics Project to validate item and scale embeddings.
Recruitment	We did not recruit participants for this study. We used data from existing data sets.
Ethics oversight	*Identify the organization(s) that approved the study protocol.*

Note that full information on the approval of the study protocol must also be provided in the manuscript.

Field-specific reporting

Please select the one below that is the best fit for your research. If you are not sure, read the appropriate sections before making your selection.

☐ Life sciences ☒ Behavioural & social sciences ☐ Ecological, evolutionary & environmental sciences

For a reference copy of the document with all sections, see nature.com/documents/nr-reporting-summary-flat.pdf

Behavioural & social sciences study design

All studies must disclose on these points even when the disclosure is negative.

Study description	The study is quantiative in nature. It involved using embeddings of linguistic data from personality measures that are publicly available and comparison to aggregate results based on human data.
Research sample	For this study we used existing datasets and did not recruit human research participants. The descriptions and sources are provided in the methods section. Data used in our work (personality measures and data from IPIP and Rosenbusch et al., 2020).
Sampling strategy	We included all available data concerning items (International Personality Item Pool and Data of Rosenbusch et al., 2020) and available construct definitions (American Psychological Association Dictionary) in our analyses. In addition, we used personality data from existing data repositories (Johnson, 2014, 2020; Open Psychometrics Project).
Data collection	We did not directly collect data from human research participants but analyzed data from existing data sets.
Timing	We included data that was available as of July 1, 2024.
Data exclusions	For our validation analyses (e.g., comparison of psychometric characteristics based on LLMs to those of human data) we selected personality data from English-speaking population only (Johnson, 2014; Open Psychometrics Project) given our focus on English personality items (IPIP) and definitions (APA).
Non-participation	In this study we did not directly collect data from human research participants and therefore our protocol does not describe or address non-participation.
Randomization	Participants were not allocated to experimental groups.

Reporting for specific materials, systems and methods

We require information from authors about some types of materials, experimental systems and methods used in many studies. Here, indicate whether each material, system or method listed is relevant to your study. If you are not sure if a list item applies to your research, read the appropriate section before selecting a response.

Materials & experimental systems

n/a	Involved in the study
☒	☐ Antibodies
☒	☐ Eukaryotic cell lines
☒	☐ Palaeontology and archaeology
☒	☐ Animals and other organisms
☒	☐ Clinical data
☒	☐ Dual use research of concern
☒	☐ Plants

Methods

n/a	Involved in the study
☒	☐ ChIP-seq
☒	☐ Flow cytometry
☒	☐ MRI-based neuroimaging

Plants

Seed stocks	none
Novel plant genotypes	none
Authentication	none
